# New Treatments for Systemic Lupus Erythematosus on the Horizon: Targeting Plasmacytoid Dendritic Cells to Inhibit Cytokine Production

**DOI:** 10.4172/2155-9899.1000534

**Published:** 2017-12-20

**Authors:** Laura M. Davison, Trine N. Jorgensen

**Affiliations:** Department of Immunology, Lerner Research Institute, Cleveland Clinic Foundation, Cleveland, Ohio, USA

**Keywords:** Systemic lupus erythematosus, Plasmacytoid dendritic cells, Interferon alpha, Autoimmune disorder, Autoimmunity

## Abstract

Patients with systemic lupus erythematosus (SLE) often have elevated levels of type I interferon (IFN, particularly IFNα), a cytokine that can drive many of the symptoms associated with this autoimmune disorder. Additionally, the presence of autoantibody-secreting plasma cells contributes to the systemic inflammation observed in SLE and IFNα supports the survival of these cells. Current therapies for SLE are limited to broad immunosuppression or B cell-targeting antibody-mediated depletion strategies, which do not eliminate autoantibody-secreting plasma cells. Recent clinical trials testing the efficacy of IFNα neutralization in SLE have delivered disappointing results, with primary endpoints not being met or with minimal improvements, while studies evaluating antibody therapy targeting the type I IFN receptor was more successful and is currently being tested in phase III clinical studies. As many studies have supported the idea that plasmacytoid dendritic cells (pDCs) are the main source of IFNα in SLE, specifically targeting pDCs in SLE represents a new therapeutic option. Murine models suggest pDC ablation effectively ameliorates or reduces lupus-like disease development in spontaneous models of lupus and pre-clinical and phase I clinical trials support the safety of such a therapy in humans. Here we review animal studies and the current status of clinical trials targeting IFNα, type I interferon receptor and pDCs in SLE.

## Introduction

**Figure 1:**
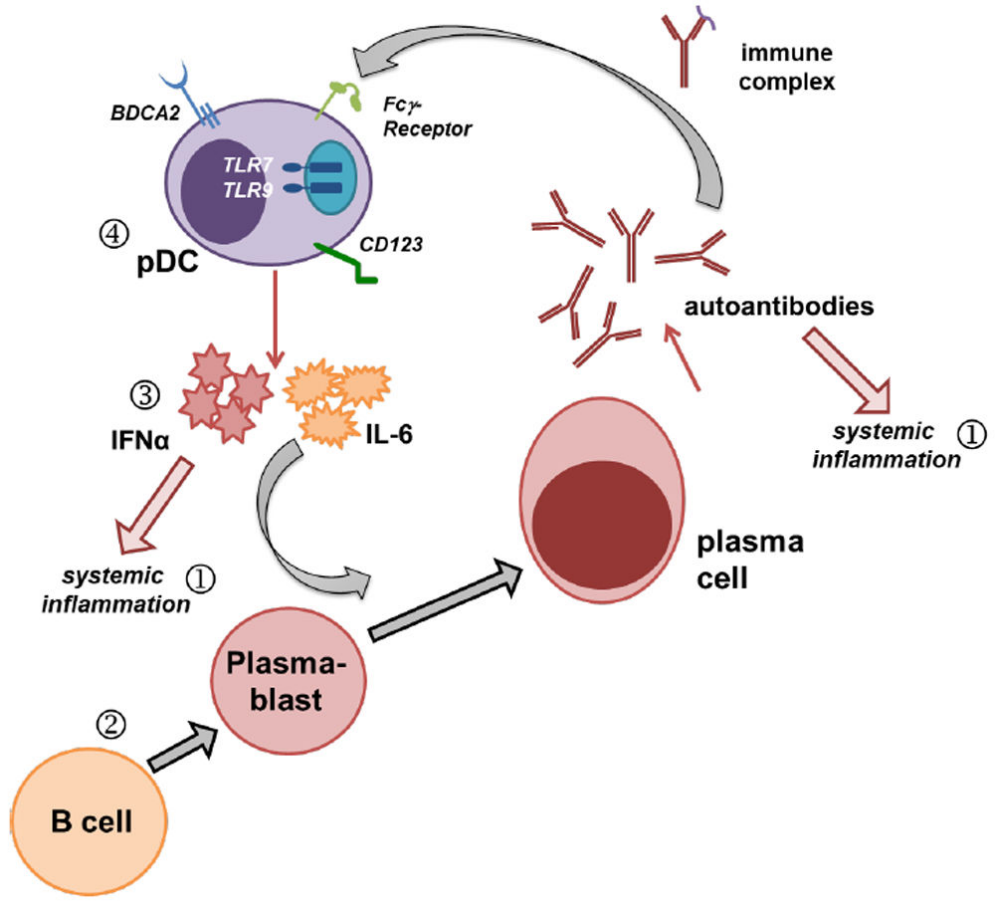
Schematic representation of the function of IFNα and pDCs in SLE and therapeutic targets currently approved or under investigation for treating SLE patients. Activated pDCs produce cytokines such as IFNα and IL-6. IFNα and IL-6 promote plasmablast expansion and maturation into antibody-secreting plasma cells. In the context of SLE, these antibodies recognize self-antigens of predominantly nuclear origin. In the circulation, antinuclear autoantibodies bind their cognate antigen to form immune complexes. pDCs express Fcγ receptors, which can mediate uptake of these immune complexes and facilitate TLR7 or TLR9 crosslinking leading to additional IFNα production. This model highlights a number of points at which therapeutics have attempted to break the cycle. (1) Current therapeutic options include antiinflammatory drugs to manage symptoms and (2) B cell depletion or inhibition of B cell survival factors removes precursors to autoantibody-secreting plasma cells. (3) Ongoing clinical trials are assessing the efficacy of IFNα neutralizing antibodies or IFNAR blocking, (4) as well as multiple different pDC targeting molecules including anti-BDCA2 and anti-CD123.

Systemic lupus erythematosus (SLE) is an autoimmune disorder with wide-ranging clinical manifestations. Although SLE varies patient to patient, one of the clinical hallmarks is the presence of autoantibodies and immune complexes, which contain host-derived nucleic acids, in the circulation [1-3]. These immune complexes stimulate type I interferon-alpha (IFNα) and interleukin 6 (IL-6) production from plasmacytoid dendritic cells (pDCs) *via* toll-like receptor 7 (TLR7) and TLR9. IFNα and IL-6 both, in turn, support auto-reactive B cells and plasmablast expansion, subsequently driving the differentiation and accumulation of auto-antibody producing plasma cells [4]. In the last decade, B cell depleting antibodies such as anti-CD20 (e.g. Rituximab, Ocrelizumab, Ofatumumab) and drugs that inhibit B cell activating factors (e.g. Belimumab, BLyS inhibition; Atacicept, BLyS/APRIL inhibition) have been introduced as new therapies for SLE patients [5] (see Figure 1 for overview of current therapeutic targets). B cell depletion or inhibition of B cell maturation has proven useful in cases of severe disease and occasionally in patients who do not respond to global immune suppression with steroids or non-steroidal anti-inflammatory drugs: the current “standard of care”. Although B cell depleting therapies offer a more directed treatment with fewer side effects than the traditional standard of care options, patients still experience serious adverse events. The main concern is an elevated risk of severe infection (upper respiratory tract and urinary tract infections, as well as influenza) in addition to patients experiencing headaches and joint pain [5]. Moreover, current B-cell targeting strategies fail to target plasma cells [6,7] and do not reduce circulating levels of autoantibodies and immune complexes (reviewed in [8]). Thus, there remains a desperate need for new SLE-specific treatment options.

## IFNα Targeting Therapies

IFNα has become an intriguing therapeutic target in SLE [9,10], given the prominent presence of elevated IFNα levels in SLE patients (reviewed in [11]) and the fact that many patients carry mutations in genes regulating type I IFN production or signaling [12]. Over 50% of patients [13] exhibit what is known as an “IFN signature”: increased expression of IFN-inducible genes, as measured in peripheral blood mononuclear cells [14,15]. The main source of IFNα in SLE is pDCs [16]. Although the initial cause of activation of pDCs remains unknown, both viral stimuli [17] and genetic contributions have been suggested [18]. Regardless, activating pDCs initiates a positive feedback loop where IFNα causes pDCs to become more responsive to the self-DNA-containing immune complexes and produce yet more IFNα [19]. Early studies in a number of mouse models of lupus (such as NZB, B6.Nba2, NZM2328, and 564Igi transgenics) showed that interference with IFNα signaling effectively prevented disease development [20-23]. Furthermore, elevating endogenous IFNα levels in lupus-prone animals resulted in accelerated or worsened disease [24,25], suggesting a causative effect of this cytokine.

Based on these studies, neutralizing IFNα or inhibiting IFNα signaling by blocking the type I IFN receptor (IFNAR) has been explored for therapeutic options. Rontalizumab and sifalimumab are anti-IFNα drugs that have been tested in SLE patients. Both drugs completed phase II studies with surprisingly low impact. Treatment with rontalizumab did not meet the primary endpoint, although a subgroup analysis showed some efficacy in patients with low IFN signature [26]. Sifalimumab completed phase IIb clinical trials early in 2015. A summary report [27] describes the primary endpoint as being met, with 56.5-59.8% of patients treated with sifalimumab experiencing improved SLE Disease Activity Index 2000 (SLEDAI-2K) score. However, 48.6% of patients receiving placebo in this study also showed improved SLEDAI-2K score at the end of the trial, significantly dampening the enthusiasm. It should also be noted that while the IFN signature was reduced following treatment with sifalimumab, serum autoantibody levels did not decrease [27]. Neither rontalizumab nor sifalimumab has moved forward to phase III clinical trials and rontalizumab was discontinued in 2014 [28].

Given the highly fluctuating levels of IFNα due to continuous exposure to virus and bacteria, it is maybe not surprising that treatment with a fixed concentration of anti-IFNα antibodies did not succeed in patients with high IFNα levels. As this patient group is mostly at risk for organ-specific pathogenesis and mostly in need of better therapeutic options, focus was shifted to investigating the effect of anti-IFNAR specific antibodies. As mentioned, IFNAR-deficient lupus-prone mice were known to be protected from disease development and recently it was shown that lupus-prone 564Igi and NZBWF1 mice treated with an anti-IFNAR antibody resulted in a significantly reduced IFN signature and reduced lupus-associated CNS pathogenesis [29]. Levels of serum autoantibody levels were not tested in this study. Anifrolumab is a human anti-IFNAR molecule that binds the IFNAR1 subunit, inhibiting type I IFN signaling [30]. Phase IIb clinical trials tested the effect of anifrolumab in 307 SLE patients. The studies were completed in 2015 and data analyses showed that both primary and secondary end points were met: 28.8-34.4% of patients showed improved SLEDAI-2K scores versus 17.6% of placebo-treated patients, 58.3-63% of anifrolumab-treated patients saw >50% improvement in skin versus 30.8% in the placebo group, and 64.6-69.6% of anifrolumab-treated patients experienced a >50% decrease in swollen and tender joints versus 48.6% of placebo-treated patients [30]. Interestingly, the effect was predominantly driven by response to treatment in patients with high IFN levels, suggesting that this population might be a primary target. Herpes zoster infection was the main adverse effect observed, however it was noted that all patients responded to anti-viral therapy to manage this occurrence. A phase III clinical trial with a target enrollment of 360 patients is currently under way (Clinicaltrials.gov; NCT02446899).

## pDC Targeting Therapies

The newest exciting treatment option on the horizon includes targeting pDCs directly. Although pDCs are a rare population of cells, they have the most potent capacity to produce IFNα of any IFN-producing cell [31,32]. pDCs have therefore long been suspected to be pathogenic in SLE. Although only indirect evidence is available implicating pDC pathogenesis in human disease [33-36], recent studies in mouse models of SLE [37,38] provide more concrete evidence. These two studies independently showed that early depletion of pDCs in spontaneous models of lupus ameliorates, or significantly reduces, subsequent development of lupus-like disease. In addition, lupus-prone mice haplodeficient for the pDC-specific Tcf4 transcription factor expressed fewer pDCs and developed reduced symptoms of disease, particularly affecting germinal center reaction and autoantibody production [39]. Although it is clear that IFNα levels drop immediately following initial depletion of pDCs [38], studies reporting on the long-term effects on IFNα production and the type I IFN signature have not been published.

Current clinical studies targeting pDCs focus on the association between BDCA2 expression and the cells’ capacity to produce IFNα [34]. Studies carried out in cynomologus monkeys showed that anti- BDCA2 antibodies inhibited IFNα-production by pDCs and significantly reduced the interferon signature [10,40]. A humanized anti-BDCA2 antibody, BIIB059, capable of binding to BDCA2 and driving receptor internalization resulting in reduced IFNα production is currently being tested. Safety and tolerability studies have been reported as acceptable [41], and a phase II clinical study is underway to establish efficacy in cutaneous lupus erythematosus (Clinicaltrials.gov; NCT02847598). Early analyses from this study supported a reduced type I IFN signature in blood and skin and reduced cell infiltration in cutaneous lesions [42]. Although further studies are needed to establish efficacy compared with currently available therapies (immunosuppression, anti-B cell therapies, anti-IFNAR therapies), these studies suggest that targeting IFNα production by pDCs may be highly effective and more specific leading to fewer adverse events [41,42].

Although anti-BDCA2 treatment results in reduced IFNα and type I IFN signature, it remains unclear if other pDC-derived inflammatory cytokines such as IL-6 (supporting autoreactive B cells) are also affected. Studies targeting IL-6 in mouse models of lupus have shown significant effects resulting in reduced serum autoantibody levels and diminished renal pathology [43-45]. Despite these pre-clinical results, a small, short-term phase II clinical trial testing the effect of anti-IL-6 monoclonal antibodies (PF-04236921) in SLE patients did not meet the primary endpoints; although several parameters, including levels of anti-dsDNA autoantibodies and disease flares, were reduced in patients treated with a low-dose of antibody [46]. It remains to be tested if a combination treatment including anti-IL-6 and either anti- IFNα/IFNAR or anti-BDCA2 antibodies would be effective in reducing both serum autoantibody levels and unrelated IFNα-dependent pathogenic events.

Finally, targeting the differentiation or survival of pDCs, diminishing cell numbers rather than function, represents an alternative mechanism currently being explored. CD123 (the IL-3Rα chain) is expressed in high levels on human pDCs and IL-3 signaling is critical for pDC survival. An anti-CD123 antibody has been tested for use in acute myeloid leukemia, demonstrating its safe use in humans (Clinicaltrials.gov; NCT01632852). It has been proposed as effective in SLE as well [10] given that the antibody depletes pDCs, inhibits TLR7- and TLR9-stimulated IFNα production, and eliminates the IFN signature ex vivo in SLE patients.

## Summary and Concluding Remarks

SLE is an autoimmune disorder that predominantly affects women (9:1 over men) and often manifests during childbearing years. There is currently no cure. Traditionally, non-steroidal anti-inflammatory drugs and broad immunosuppression have been used to treat symptoms. B cell depleting therapies help in some cases by temporarily reducing the precursors to autoantibody-secreting plasma cells, but the need remains for a therapy targeting key players in SLE. Recent clinical trials inhibiting IFNα-signaling or neutralizing IFNα are promising, but patients still experience adverse events. The newest development indicates that targeting pDCs, for depletion or to inhibit cytokine production, in SLE is a more specific way to interfere with the positive feed-back loop of IFNα production and signaling, in addition to eliminating the cytokines that support autoantibody-secreting plasma cells. The results of current clinical trials are eagerly anticipated along with future studies addressing the effectiveness of combination therapies concomitantly targeting a multitude of clinical manifestations of SLE.

## Abbreviations

IFN: Interferon
IFNAR: Type I Interferon Receptor
pDC: Plasmacytoid Dendritic Cell
SLE: Systemic Lupus Erythematosus
TLR: Toll Like Receptor
